# Use of the Internet for Sexual Health Among Sexually Experienced Persons Aged 16 to 44 Years: Evidence from a Nationally Representative Survey of the British Population

**DOI:** 10.2196/jmir.4373

**Published:** 2016-01-20

**Authors:** Catherine RH Aicken, Claudia S Estcourt, Anne M Johnson, Pam Sonnenberg, Kaye Wellings, Catherine H Mercer

**Affiliations:** ^1^ Research Department of Infection and Population Health Institute of Epidemiology and Healthcare University College London London United Kingdom; ^2^ Blizard Institute, Centre for Immunology and Infectious Diseases Barts and the London School of Medicine and Dentistry Queen Mary University of London London United Kingdom; ^3^ Centre for Sexual and Reproductive Health Research Department of Social and Environmental Health Research London School of Hygiene and Tropical Medicine London United Kingdom

**Keywords:** sexual health, sexually transmitted diseases, contraception, health care-seeking behavior, Internet, eHealth, surveys, information-seeking behavior

## Abstract

**Background:**

Those who go online regarding their sexual health are potential users of new Internet-based sexual health interventions. Understanding the size and characteristics of this population is important in informing intervention design and delivery.

**Objective:**

We aimed to estimate the prevalence in Britain of recent use of the Internet for key sexual health reasons (for chlamydia testing, human immunodeficiency virus [HIV] testing, sexually transmitted infection [STI] treatment, condoms/contraceptives, and help/advice with one’s sex life) and to identify associated sociodemographic and behavioral factors.

**Methods:**

Complex survey analysis of data from 8926 sexually experienced persons aged 16-44 years in a 2010-2012 probability survey of Britain’s resident population. Prevalence of recent (past year) use of Internet sources for key sexual health reasons was estimated. Factors associated with use of information/support websites were identified using logistic regression to calculate age-adjusted odds ratios (AORs).

**Results:**

Recent Internet use for chlamydia/HIV testing or STI treatment (combined) was very low (men: 0.31%; women: 0.16%), whereas 2.35% of men and 0.51% of women reported obtaining condoms/contraceptives online. Additionally, 4.49% of men and 4.57% of women reported recent use of information/support websites for advice/help with their sex lives. Prevalence declined with age (men 16-24 years: 7.7%; 35-44 years: 1.84%, *P*<.001; women 16-24 years: 7.8%; 35-44 years: 1.84%, *P*<.001). Use of information/support websites was strongly associated with men’s higher socioeconomic status (managerial/professional vs semiroutine/routine: AOR 1.93, 95% CI 1.27-2.93, *P*<.001). Despite no overall association with area-level deprivation, those in densely populated urban areas were more likely to report use of information/support websites than those living in rural areas (men: AOR 3.38, 95% CI 1.68-6.77, *P*<.001; women: AOR 2.51, 95% CI 1.34-4.70, *P*<.001). No statistically significant association was observed with number of sex partners reported after age adjustment, but use was more common among men reporting same-sex partners (last 5 years: AOR 2.44, 95% CI 1.27-4.70), women reporting sex with multiple partners without condoms (last year: AOR 1.90, 95% CI 1.11-3.26), and, among both sexes, reporting seeking sex online (last year, men: AOR 1.80, 95% CI 1.16-2.79; women: AOR 3.00, 95% CI 1.76-5.13). No association was observed with reporting STI diagnosis/es (last 5 years) or (after age adjustment) recent use of any STI service or non-Internet sexual health seeking.

**Conclusions:**

A minority in Britain used the Internet for the sexual health reasons examined. Use of information/support websites was reported by those at greater STI risk, including younger people, indicating that demand for online STI services, and Internet-based sexual health interventions in general, may increase over time in this and subsequent cohorts. However, the impact on health inequalities needs addressing during design and evaluation of online sexual health interventions so that they maximize public health benefit.

## Introduction

Sexual health is increasingly recognized as encompassing physical, mental, and emotional well-being in relation to sexuality and sexual relationships, and freedom from coercion [1]. In Britain, and globally, there has been an expansion in online sexual health services [2-5]. As well as providing information, these services take advantage of the interactive potential of the Internet, such as for sexual health promotion [6], to aid contraceptive choices [7], or for individual counseling via Web chat [8,9]. Condoms and contraceptives are purchasable online from Internet vendors and pharmacies. Regarding sexually transmitted infections (STIs), England’s National Chlamydia Screening Programme (NCSP) provides free, Internet-ordered home-sampling kits to those aged 16-24 years in many localities [5]. Privately provided Internet-ordered STI and human immunodeficiency virus (HIV) testing and STI treatment services are increasingly available, although they have been poorly regulated and of variable quality [3,5]. Recently, the British government legalized HIV home tests, which have been available for purchase online since 2015 [10].

Internet access is now nearly universal among people of reproductive age in the United Kingdom (98% aged 16-34 years, 93% aged 35-44 years in 2013) and more than one-third regularly uses the Internet to find information on health-related issues [11]. Although new Internet-based sexual health services continue to be developed [12-15], the number and characteristics of people who use currently available online sexual health services in Britain are unknown. To inform the design and delivery of new online sexual health interventions and services, we need to understand the demographic and behavioral characteristics of existing users. This will help inform whether Internet-based services could reach populations that underutilize conventional sexual health services relative to their need for sexual health care. This may include people at elevated risk of STI, such as young people (aged 16-24 years), people of black ethnic origins, men who have sex with men (MSM) [16], those who report multiple sexual partners, those living in deprived areas [17], and sexually active people who report no recent sexual health care use. This evidence is necessary for estimating the likely impact of online services which are currently being developed, and for informing the targeting of these services to maximize public health benefit. This study aims to fill this evidence gap by providing evidence of the British population’s use of existing Internet-based sexual health services and the population who report using them. We conjectured that those reporting use of the Internet for these reasons might represent a population likely to take up online sexual health services that are currently being developed. Our study’s focus was on the year before the survey interview to provide a contemporary picture in a rapidly changing field.

Specific objectives were (1) to estimate the prevalence of reporting recent (in the previous year) use of the Internet as a source of chlamydia testing, HIV testing, STI treatment, condoms/contraceptive supplies, and help/advice with one’s sex life from information/support websites among sexually experienced men and women; (2) to describe the population reporting this; and (3) to estimate the proportions reporting a preference for online sexual health care.

## Methods

### Natsal-3 Survey Design and Administration

Britain’s third National Survey of Sexual Attitudes and Lifestyles (Natsal-3 [18,19]) is a probability sample survey conducted between 2010 and 2012 among the British resident population aged 16 to 74 years (N=15,162). Natsal-3 asked detailed demographic and behavioral questions and a number of questions about sources used for various types of sexual health care and advice/help with one’s sex life (including the Internet). Detailed methods have been reported elsewhere; briefly, Natsal-3 used a multistage, clustered, and stratified probability sample design with a boost sample of those aged 16 to 34 years [18,19]. An interviewer visited each selected household and randomly selected one person in the eligible age range to participate, with oral informed consent. Participants completed the survey using a mixture of computer-assisted personal interview (CAPI) conducted face-to-face and computer-assisted self-interview (CASI) for the more sensitive questions [18,19]. Natsal-3 achieved an overall response rate of 57.7% and a cooperation rate (of eligible addresses contacted) of 65.8% [18,19].

The full survey is available online [20]. Variables used in this study were based on self-reported responses to closed-ended survey questions, except Index of Multiple Deprivation (IMD) quintile [21] and Output Area Classification (OAC) 2011 supergroup (OAC 2011 categorizes census output areas into 8 supergroups based on population characteristics) [22,23]. These were added to the dataset according to participants’ postcodes. National Statistics Socio-Economic Classification (NS-SEC) was derived from responses to standard questions [24].

### Population of Interest: Sexually Experienced Persons Aged 16 to 44 Years

Several survey questions relevant to these analyses were not asked to participants aged 45 years and older. Therefore, the denominator for this study was limited to those aged 16 to 44 years, the age group in which most STI diagnoses occur [16], and which approximates women’s reproductive age. We further limited the denominator to sexually experienced people, defined as those who reported ever having had any opposite- or same-sex sexual partners, because they are most likely to require sexual health services.

### Outcome Variables

Outcome variables for this study included reported use of Internet services for key sexual health reasons (Table 1) and reporting the Internet as a preferred source of contraception, or for STI treatment/diagnosis if an STI was suspected (Table 2). The wording of these survey questions is described in Tables 1 and 2. Of specific relevance to the question about help/advice with one’s sex life (first question in Table 1), shortly before this question, participants were presented with the following broad definition of *sex life*: “An individual’s sex life includes their sexual thoughts, sexual feelings, sexual activity and sexual relationships.”

For timeframe, the question on sources of contraceptive supplies referred to the past year. Questions on HIV testing, chlamydia testing, and STI treatment referred to the last occurrence. For comparability, only participants who indicated that this last occurrence was in the previous year (determined from responses to other survey questions) were included as reporting these behaviors.

### Explanatory Variables

We had the following categories of explanatory variables: participants’ sociodemographics, Internet access, area-level measures, sexual behavior (in the past year and past 5 years), sexual health care use, and STI diagnosis. Variables for sexual behavior and service use were selected to match the timeframe of the primary outcome variable (the year before the survey interview). Some variables corresponding to the 5 years before the interview were included (having had same-sex partners, number of sexual partners, sexual health clinic attendance, and STI diagnosis) to reflect greater variability in certain behaviors in the population over this longer period [25].

### Statistical Methods

Data were analyzed using the complex survey functions of Stata 12 to take account of clustering, stratification, and weighting of the Natsal-3 sample. Weights were applied to adjust for unequal probabilities of selection for participation in the survey. All analyses were conducted separately by sex. Participants with missing data for a given variable were excluded from analyses using this variable because item nonresponse in Natsal-3 was low (typically less than 0.5% in the CAPI and 1%-3% in the CASI) [18].

Logistic regression was used to obtain crude odds ratios to compare estimates of the odds of reporting use of information/support websites for advice/help with one’s sex life, by each explanatory variable. Multivariable logistic regression was used, adjusting only for age, as a potential confounder of associations with NS-SEC code, which contained a “full-time student” category; OAC 2011, which was based on population characteristics including age; recent STI diagnosis; and sexual behavior variables because young people report greater numbers of recent and new sexual partners than older adults [25].

The observed low prevalences of other outcome variables meant that it was not possible to explore their associated factors. Statistical significance was considered as *P*<.05 for all analyses.

### Ethical Approval

The Natsal-3 study was approved by the Oxfordshire Research Ethics Committee A (Ref: 10/H0604/27).

## Results

### Prevalence of Reported Recent Use of the Internet for Selected Sexual Health Reasons

**Figure 1:**
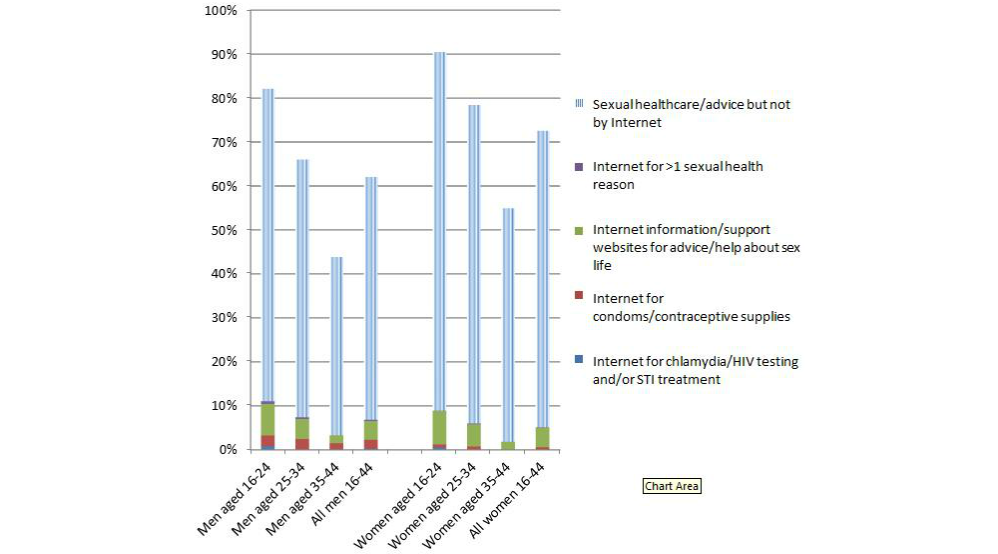
Percentage reporting seeking sexual health care and advice/help with one’s sex life in the previous year, and specifically using the Internet to do so, among sexually experienced persons aged 16-44 years by gender and age group.

Among sexually experienced persons aged 16 to 44 years, Internet use for chlamydia testing, HIV testing, or STI treatment (combined) in the previous year was reported by 0.31% (12/3702) men and 0.16% (6/3716) women (Figure 1). (Note: numerators and denominators are weighted and rounded to the nearest integer so may be subject to rounding errors.) Mostly this was chlamydia testing. No one in the sample reported Internet treatment for STIs other than chlamydia. Also, no one aged 35 to 44 years reported using the Internet for chlamydia testing, HIV testing, or STI treatment. Use of Internet sources of contraception/condoms in the past year was a little more common, especially among men (men: 2.35%, 87/3702; women: 0.51%, 19/3716). (Participants were not asked which method they obtained online, but it is likely that this was mostly condoms: 114 of 122 men and women reporting obtaining contraceptive supplies online in the past year reported use of male [n=113] and/or female [n=2] condoms in this period.) Use of information and support websites for advice/help with one’s sex life in the past year was more common still, reported by 4.49% (166/3702) men and 4.57% (170/3716) women. Overall, use of the Internet for any of these sexual health reasons in the past year was reported by 6.85% men (95% CI 6.02-7.78) and 5.15% women (95% CI 4.50-5.89). In contrast, 60.2% men (95% CI 58.2-62.1) and 71.7% women (95% CI 70.2-73.2) reported use of non-Internet sources of sexual health care or advice/help with their sex lives, in the past year. (We defined this as GUM clinic attendance; use of non-Internet sources of chlamydia/HIV testing, STI treatment, or condoms/contraceptive supplies; or non-Internet sources of advice/help with one’s sex life, excluding self-help and friends/family, in the past year.)

**Table 1:** Details of the Natsal-3 survey questions used as outcome variables in these analyses of sexually experienced persons aged 16 to 44 years (unweighted N=8926, weighted N=7400).

Question wording	Timeframe; number of responses permitted	Response options	Respondents eligible for each survey question	Number eligible for each question, unweighted (weighted)
Have you sought help or advice regarding your sex life from any of the following sources in the last year?	During previous year; multiple responses	Information and support sites on the Internet;^a^ family member/friend; self-help books/information leaflets; self-help groups; helpline; GP/family doctor; sexual health/GUM/STI clinic; psychiatrist or psychologist; relationship counsellor; other type of clinic or doctor; have not sought any help	Entire sample of the current study	8926 (7400)
Have you got contraception from any of these sources in the last year?	During previous year; multiple responses	Internet website;^a^ a doctor or nurse at your GP’s surgery; sexual health clinic (GUM clinic); family planning clinic / contraceptive clinic / reproductive health clinic; NHS antenatal clinic / midwife; private doctor or clinic; youth advisory clinic (eg, Brook clinic); pharmacy/chemist; supplies from school/college/university services; over the counter at a petrol station/supermarket/other shop; vending machine; mail order; hospital accident and emergency (A&E) department; any other type of place (please say where); I have not got contraception in the last year	Those reporting use of any contraceptive method^b^ in the last year	7182 (5862)
When you were last tested for chlamydia, where were you offered the test?	Last occurrence; single response	Internet;^a^ GP surgery; sexual health clinic (GUM clinic); NHS family planning clinic / contraceptive clinic / reproductive health clinic; antenatal clinic/midwife; private non-NHS clinics or doctor; youth advisory clinic (eg, Brook Clinic); School/college/university; termination of pregnancy (abortion) clinic; hospital accident and emergency (A&E) department; pharmacy/chemist; other non-health care place (eg, youth club, festival, bar); somewhere else	Those reporting chlamydia testing in the last year	2387 (1545)
Where were you tested? (the last HIV test if more than one)	Last occurrence; single response	Internet site offering postal kit;^a^ GP surgery; sexual health clinic (GUM clinic); NHS family planning clinic / contraceptive clinic / reproductive health clinic; antenatal clinic / midwife; private non-NHS clinic or doctor; youth advisory clinic (eg, Brook clinic); termination of pregnancy (abortion) clinic; hospital accident and emergency (A&E) department; somewhere else	Those reporting HIV testing in the last year	802 (562)
Where were you last treated for [STI^c^]?	Last occurrence; single response	Internet site offering treatment;^a^ GP surgery; sexual health clinic (GUM clinic); NHS family planning clinic / contraceptive clinic / reproductive health clinic; antenatal clinic / midwife; private non-NHS clinic or doctor; pharmacy/chemist; youth advisory clinic (eg, Brook clinic); termination of pregnancy (abortion) clinic; hospital accident and emergency (A&E) department; somewhere else	Those reporting having been told by a doctor / health professional that they had an STI in the last year	178 (117)

^a^ Internet response options.

^b^ Including condoms.

^c^ Separate questions were asked about the following infections: chlamydia; gonorrhea; genital warts; syphilis; *Trichomonas vaginalis*; genital herpes; nonspecific urethritis (NSU) or nongonococcal urethritis (NGU).

**Table 2:** Natsal-3 survey questions about preferred sources of sexual health care.

Question wording^a^	Response options	Respondents eligible for each survey question	Number eligible for each question, unweighted (weighted)
If you thought that you might have an infection that is transmitted by sex, where would you *first* go to seek diagnosis and/or treatment?	Internet site offering treatment;^b^ GP surgery; sexual health clinic (GUM clinic); NHS Family planning clinic/contraceptive clinic/reproductive health clinic; NHS antenatal clinic/midwife; private non-NHS clinic or doctor; pharmacy/chemist; youth advisory clinic (eg, Brook clinic); hospital accident and emergency (A&E) department; somewhere else	Those reporting any lifetime sexual partners	8858 (7338)
If all of these different types of service were available in your area and easy to get to, which one would *you* prefer to get contraception from?	NHS or Department of Health website;^b^ a doctor or Nurse at your GP’s surgery; sexual health clinic (GUM clinic); family planning clinic / contraceptive clinic / reproductive health clinic; youth advisory clinic (eg, Brook clinic); pharmacy/chemist; none of these; not needed	Those reporting use of any method in the last year	6909 (5524)

^a^ Use of italics reflects emphasis given in the survey. One response could be selected at each question.

^b^ Internet response options.

### Associations with Reporting Use of Information and Support Websites for Advice/Help with One’s Sex Life

#### Sociodemographic Factors

Mean age of men and women reporting use of Internet information/support websites for advice/help with their sex life (based on the first question described in Table 1 and hereon referred to as “Internet information/support” for brevity) was 25.9 years (SD 7.5) and 26.9 years (SD 8.8), respectively, in this sample aged 16 to 44 years. Those not reporting this were on average older (men: 31.0 years, SD 8.0; women: 31.3 years, SD 9.7). The prevalence of reporting use of Internet information/support declined steeply with increasing age among both sexes (7.7% men, 7.8% women aged 16-24 years to 1.84% men, 1.84% women aged 35-44, both *P*<.001). Tables 3 and 4 present univariate and age-adjusted analyses among men and women, respectively.

**Table 3:** Variation in the prevalence and odds of reporting recent (past year) use of information/support websites for advice/help with one’s sex life (Internet information/support) among sexually experienced men aged 16 to 44 years.^a^

Variable			N, unweighted (weighted)	Prevalence (95% CI)	OR (95% CI)	*P*	AOR (95% CI)	*P*
**Sociodemographics**								
	**Age (years)**					<.001		—
		16-24	1361 (994)	7.7% (6.3-9.4)	1		—	
		25-34	1451 (1299)	4.93% (3.90-6.23)	0.62 (0.45-0.86)		—	
		35-44	784 (1383)	1.84% (1.12-3.02)	0.22 (0.13-0.39)		—	
	**Ethnic group**					.007		.004
		White	3134 (3118)	4.01% (3.39-4.75)	1		1	
		Asian/Asian British	190 (270)	6.9% (4.0-11.6)	1.77 (0.98-3.21)		2.11 (1.16-3.84)	
		Black/black British	126 (140)	7.8% (3.7-15.4)	2.01 (0.92-4.42)		2.11 (0.93-4.81)	
		Mixed/Chinese/other	108 (110)	9.4% (5.1-16.8)	2.49 (1.26-4.93)		2.2 (1.13-4.26)	
	**Education level** ^b^					<.001		<.001
		No academic qualifications	252 (275)	0.8% (0.3-2.5)	0.60 (0.18-2.00)		0.65 (0.20-2.18)	
		Academic qualifications typically gained at age 16	880 (912)	1.4% (0.8-2.3)	1		1	
		Studying for/attained further academic qualifications	2354 (2419)	6.05% (5.13-7.13)	4.57 (2.68-7.78)		3.79 (2.20-6.51)	
	**Socioeconomic status** ^c^					<.001		.001
		Managerial/professional	1060 (1262)	4.53% (3.42-5.98)	1.46 (0.97-2.19)		1.93 (1.27-2.93)	
		Intermediate	509 (554)	3.0% (1.8-4.8)	0.94 (0.53-1.66)		1.16 (0.64-2.08)	
		Semiroutine/routine	1321 (1300)	3.15% (2.40-4.11)	1		1	
		No job	122 (99)	1.6% (0.4-6.4)	0.48 (0.11-2.08)		0.33 (0.08-1.42)	
		Full-time student	574 (452)	11.1% (8.5-14.5)	3.85 (2.53-5.86)		1.95 (1.14-3.34)	
**Internet access**								
	**Access to Internet at home**					.02		.02
		Yes	3327 (3442)	4.73% (4.06-5.51)	1		1	
		No	267 (232)	1.5% (0.6-3.9)	0.30 (0.11-0.82)		0.31 (0.11-0.84)	
**Area-level measures**								
	**Deprivation** ^d^					.51		.24
		1 (least deprived)	642 (658)	5.7% (4.2-7.7)	1		1	
		2	653 (699)	4.3% (3.1-6.0)	0.74 (0.46-1.20)		0.71 (0.44-1.14)	
		3	690 (720)	4.6% (3.3-6.5)	0.81 (0.50-1.30)		0.76 (0.47-1.23)	
		4	774 (823)	4.3% (2.9-6.4)	0.75 (0.45-1.26)		0.69 (0.41-1.15)	
		5 (most deprived)	837 (776)	3.8% (2.7-5.3)	0.66 (0.41-1.06)		0.58 (0.36-0.93)	
	**Output Area Classification 2011**					<.001		<.001
		1: “Rural residents”	276 (294)	3.2% (1.8-5.6)	1		1	
		2: “Cosmopolitans”	302 (329)	12.5% (9.0-17.2)	4.33 (2.17-8.63)		3.38 (1.68-6.77)	
		3: “Ethnicity central”	181 (225)	5.4% (2.7-10.3)	1.71 (0.69-4.27)		1.58 (0.64-3.91)	
		4: “Multicultural metropolitans”	516 (595)	3.7% (2.3-5.7)	1.15 (0.54-2.43)		1.04 (0.49-2.22)	
		5: “Urbanites”	665 (667)	3.6% (2.4-5.3)	1.13 (0.55-2.30)		1.09 (0.53-2.24)	
		6: “Suburbanites”	587 (597)	4.5% (3.2-6.3)	1.44 (0.72-2.85)		1.30 (0.65-2.59)	
		7: “Constrained city dwellers”	331 (271)	4.1% (2.3-7.1)	1.28 (0.56-2.94)		1.06 (0.46-2.48)	
		8: “Hard-pressed living”	738 (698)	2.8% (2.0-4.0)	0.87 (0.44-1.75)		0.76 (0.38-1.52)	
**Sexual behavior, last year**								
	**Number of sexual partners**					.77		.29
		0	191 (174)	4.6% (2.4-8.6)	1.06 (0.53-2.12)		0.95 (0.48-1.89)	
		1	2320 (2612)	4.37% (3.63-5.26)	1		1	
		2+	1051 (857)	5.0% (3.7-6.6)	1.14 (0.80-1.63)		0.72 (0.48-1.08)	
	≥**1 new sexual partners**					<.001		.11
		No	2129 (2503)	3.34% (2.71-4.12)	1		1	
		Yes	1428 (1134)	7.14% (5.74-8.85)	2.22 (1.61-3.07)		1.39 (0.93-2.09)	
	**Number of sexual partners without a condom**					.12		.30
		0	862 (780)	5.9% (4.4-7.8)	1		1	
		1	2139 (2412)	4.15% (3.40-5.05)	0.69 (0.48-0.98)		0.96 (0.66-1.38)	
		≥2	523 (419)	4.5% (3.1-6.7)	0.75 (0.46-1.25)		0.69 (0.42-1.13)	
	**Seeking sexual partners online**					.004		.009
		No	3287 (3414)	4.28% (3.64-5.03)	1		1	
		Yes	306 (257)	7.9% (5.4-11.6)	1.92 (1.24-3.00)		1.80 (1.16-2.79)	
**Sexual behavior, last 5 years**								
	**Number of sexual partners**					.04		.96
		0-1	1441 (1805)	3.63% (2.82-4.66)	1		1	
		2-4	1106 (1012)	5.17% (3.98-6.70)	1.45 (0.99-2.13)		0.94 (0.63-1.41)	
		≥5	1024 (837)	5.8% (4.4-7.6)	1.64 (1.11-2.42)		0.95 (0.60-1.49)	
	≥**1 same-sex partners**					.002		.008
		No	3459 (3561)	4.32% (3.68-5.06)	1		1	
		Yes	137 (116)	10.9% (6.2-18.5)	2.71 (1.43-5.14)		2.44 (1.27-4.70)	
**Sexual health care use and STI**								
	**Non-Internet sexual health care or advice/help, last year** ^e^					.004		.42
		Yes	2391 (2223)	5.46% (4.57-6.51)	1		1	
		Not reported	1205 (1453)	3.10% (2.24-4.28)	0.55 (0.37-0.82)		0.84 (0.55-1.29)	
	**Attended STI clinic, last 5 years**					.03		.89
		Yes	861 (712)	5.9% (4.5-7.8)	1		1	
		No	2670 (2902)	4.11% (3.41-4.95)	0.68 (0.48-0.97)		0.97 (0.67-1.41)	
	**STI service use, last year** ^f^					.27		.08
		Yes	873 (703)	5.3% (3.9-7.0)	1		1	
		Not reported	2723 (2974)	4.35% (3.64-5.19)	0.82 (0.57-1.17)		1.40 (0.96-2.02)	
	**STI** ^g^ **diagnosis, last 5 years**					.68		.97
		No	3300 (3408)	4.47% (3.81-5.24)	1		1	
		Yes	257 (225)	5.0% (2.9-8.5)	1.13 (0.63-2.04)		0.99 (0.55-1.79)	

^a^ Unweighted N=3614, weighted N=3697. Denominators vary due to item nonresponse.

^b^ Denominator restricted to those aged 17 and older. No academic qualifications: left school at age 16 without passing any exams/gaining any qualifications (excludes qualifications gained at an older age); academic qualifications typically gained at age 16: left school at 16 having passed some exams/gained some qualifications (eg, English General Certificate of Secondary Education [GCSE] or equivalent); studying for or attained further academic qualifications: left school at age 17 or older.

^c^ Based on National Statistics Socioeconomic Classification (NS-SEC) code. No job: no job of ≥10 hours per week in the last 10 years.

^d^ Quintile of adjusted Index of Multiple Deprivation for Great Britain.

^e^ Defined as reporting STI clinic attendance within the last year or responses other than “Internet” for questions listed in Table 1 within the last year. Exceptions (non-Internet responses which were ignored) were (1) where participants had indicated friend, parent/relative, or partner as sources of contraceptive supplies (free-text response) and (2) where participants had selected “family member/friend,” “self-help books/information leaflets,” “self-help groups,” and “have not sought any help” as sources of advice/help about their sex life.

^f^ Defined as reporting any of: STI clinic attendance, chlamydia testing, or HIV testing within this last year.

^g^ Natsal definition of STIs excludes thrush.

A strong association was observed with education level; 1.4% of men and 2.0% of women who left school aged 16 years with General Certificates of Secondary Education (GCSEs), or equivalent qualifications, reported recent use of Internet information/support compared to 6.05% of men and 5.87% of women with, or studying for, further academic qualifications (both sexes: *P*<.001), an association which remained after age adjustment. Associations with socioeconomic status [24] followed similar trends. Men in high-status occupations were more likely to report Internet information/support than those in lower-status occupations, before and after age adjustment (managerial/professional men vs men in semiroutine/routine occupations: age-adjusted OR [AOR] 1.93, 95% CI 1.27-2.93, *P*<.001), whereas a similar finding among women reached borderline statistical significance after age adjustment. Full-time students of both genders were also more likely than those in lower-status occupations to report Internet information/support even after taking account of their younger age (men: AOR 1.95, 95% CI 1.14-3.34; women: AOR 1.93, 95% CI 1.24-3.00).

Despite associations with these individual measures of social status (education, socioeconomic status), no overall association was observed between recent use of Internet information/support and area-level deprivation [21]. Use of Internet information/support was associated with OAC 2011 supergroup. Use was high among “cosmopolitans” (residents of densely populated urban areas characterized by relatively high proportions of single people, young adults, full-time students, and high ethnic integration) [23] (men: 12.5%, 95% CI 9.0-17.2; women 11.7%, 95% CI 8.3-16.3). There was little variation between other supergroups except, among women only, slightly lower use of Internet information/support in “hard-pressed living” areas (mostly urban areas in Northern England and Wales with higher unemployment and lower proportions with higher-level qualifications than the national average) [23]. Strong associations with OAC 2011 supergroup remained after age adjustment (see Tables 3 and 4).

No overall association with ethnicity was observed among women after age adjustment, but Asian/Asian British men were more likely to report use of Internet information/support than white men (AOR 2.11, 95% CI 1.16-3.84, *P*=.004). Notably, numbers in minority ethnic groups were relatively small.

Having home Internet access was reported by 93.5% (95% CI 92.9-94.0) of sexually experienced persons aged 16 to 44 years. The minority who did not have home Internet were less likely to report use of Internet information/support than those who had (men: OR 0.30, 95% CI 0.11-0.82, *P*=.02; women: OR 0.26, 95% CI 0.11-0.58, *P*<.001) with little change after adjusting for age.

**Table 4:** Variation in the prevalence and odds of reporting recent (past year) use of Internet information/support among sexually experienced women aged 16 to 44 years.^a^

Variable			N, unweighted (weighted)	Prevalence (95% CI)	OR (95% CI)	*P*	AOR (95% CI)	*P*
**Sociodemographics**								
	**Age (years)**					<.001		—
		16-24	1713 (956)	7.8% (6.4-9.4)	1		—	
		25-34	2386 (1317)	5.28% (4.32-6.45)	0.66 (0.49-0.89)		—	
		35-44	1175 (1409)	1.84% (1.16-2.90)	0.22 (0.13-0.37)		—	
	**Ethnic group**					.02		.07
		White	4619 (3179)	4.39% (3.76-5.10)	1		1	
		Asian/Asian British	258 (220)	3.8% (2.2-6.4)	0.86 (0.49-1.52)		0.96 (0.54-1.70)	
		Black/black British	174 (136)	5.6% (3.0-10.2)	1.30 (0.67-2.52)		1.34 (0.70-2.59)	
		Mixed/Chinese/other	176 (117)	11.1% (6.1-19.3)	2.71 (1.39-5.28)		2.32 (1.20-4.50)	
	**Education level** ^b^					<.001		<.001
		No academic qualifications	372 (237)	0.6% (0.2-1.9)	0.29 (0.08-1.04)		0.28 (0.08-0.98)	
		Academic qualifications typically gained at age 16	1186 (863)	2.0% (1.3-3.1)	1		1	
		Studying for/attained further academic qualifications	3607 (2528)	5.87% (5.07-6.79)	3.05 (1.88-4.97)		2.49 (1.52-4.06)	
	**Socioeconomic status** ^c^					<.001		.06
		Managerial/professional	1526 (1202)	4.08% (3.16-5.26)	1.21 (0.79-1.85)		1.56 (1.02-2.40)	
		Intermediate	1006 (719)	3.9% (2.5-5.9)	1.14 (0.66-1.97)		1.32 (0.76-2.29)	
		Semiroutine/routine	1582 (1028)	3.39% (2.50-4.60)	1		1	
		No job	418 (285)	4.6% (2.9-7.3)	1.39 (0.78-2.46)		1.39 (0.79-2.46)	
		Full-time student	717 (429)	10.2% (7.9-13.1)	3.23 (2.14-4.89)		1.93 (1.24-3.00)	
**Internet access**								
	**Access to Internet from home**					.001		<.001
		Yes	4828 (3444)	4.84% (4.21-5.56)	1		1	
		No	443 (236)	1.3% (0.6-2.8)	0.26 (0.11-0.58)		0.23 (0.10-0.52)	
**Area-level measures**								
	**Deprivation** ^d^					.58		.35
		1 (least deprived)	847 (632)	5.5% (4.0-7.4)	1		1	
		2	952 (699)	4.4% (3.1-6.1)	0.79 (0.49-1.29)		0.78 (0.48-1.26)	
		3	1031 (739)	4.8% (3.5-6.7)	0.88 (0.55-1.41)		0.83 (0.51-1.35)	
		4	1183 (821)	4.8% (3.5-6.5)	0.87 (0.55-1.38)		0.82 (0.51-1.29)	
		5 (most deprived)	1261 (792)	3.7% (2.7-5.1)	0.68 (0.42-1.08)		0.61 (0.38-0.97)	
	**Output Area Classification 2011**					<.001		<.001
		1: “Rural residents”	414 (313)	4.0% (2.5-6.4)	1		1	
		2: “Cosmopolitans”	349 (266)	11.7% (8.3-16.3)	3.20 (1.72-5.96)		2.51 (1.34-4.70)	
		3: “Ethnicity central”	307 (257)	5.7% (3.5-9.0)	1.45 (0.72-2.91)		1.32 (0.65-2.68)	
		4: “Multicultural metropolitans”	772 (557)	5.5% (3.9-7.7)	1.40 (0.76-2.57)		1.27 (0.69-2.36)	
		5: “Urbanites”	961 (667)	4.8% (3.4-6.6)	1.20 (0.65-2.22)		1.14 (0.61-2.14)	
		6: “Suburbanites”	799 (608)	4.1% (2.8-5.8)	1.02 (0.55-1.90)		1.02 (0.55-1.92)	
		7: “Constrained city dwellers”	488 (277)	3.3% (2.0-5.4)	0.83 (0.41-1.69)		0.70 (0.35-1.42)	
		8: “Hard-pressed living”	1184 (736)	2.0% (1.3-3.1)	0.50 (0.26-0.94)		0.45 (0.24-0.86)	
**Sexual behavior, last year**								
	**Number of sexual partners**					.008		.65
		0	284 (187)	3.2% (1.7-6.0)	0.75 (0.38-1.48)		0.88 (0.45-1.73)	
		1	3829 (2825)	4.22% (3.58-4.96)	1		1	
		≥2	1113 (631)	6.9% (5.2-9.2)	1.69 (1.19-2.40)		1.18 (0.81-1.72)	
	≥**1 new sexual partners**					<.001		.11
		No	3670 (2748)	3.82% (3.19-4.56)	1		1	
		Yes	1553 (892)	7.2% (5.7-8.9)	1.95 (1.43-2.65)		1.32 (0.94-1.85)	
	**Number of partners without a condom**					<.001		.03
		0	1007 (680)	4.3% (3.1-5.8)	1		1	
		1	3620 (2635)	4.12% (3.47-4.89)	0.97 (0.67-1.40)		1.05 (0.73-1.50)	
		≥2	575 (317)	10.0% (7.1-13.9)	2.51 (1.50-4.17)		1.90 (1.11-3.26)	
	**Seeking sexual partners online**					<.001		<.001
		No	5079 (3559)	4.38% (3.78-5.06)	1		1	
		Yes	189 (116)	11.8% (7.5-18.1)	2.93 (1.74-4.94)		3.00 (1.76-5.13)	
**Sexual behavior, last 5 years**								
	**Number of sexual partners**					<.001		.18
		0-1	2649 (2116)	3.77% (3.05-4.65)	1		1	
		2-4	1630 (995)	4.6% (3.6-5.8)	1.23 (0.88-1.71)		0.88 (0.63-1.23)	
		≥5	958 (541)	8.1% (6.1-10.7)	2.25 (1.53-3.29)		1.31 (0.85-2.01)	
	≥**1 same-sex partners**					.09		.24
		No	4972 (3493)	4.47% (3.87-5.16)	1		1	
		Yes	302 (189)	7.2% (4.3-11.9)	1.65 (0.93-2.93)		1.42 (0.80-2.52)	
**Sexual health care use and STI**								
	**Non-Internet sexual health care or advice/help, last year** ^e^					<.001		.11
		Yes	4055 (2648)	5.42% (4.66-6.30)	1		1	
		Not reported	1219 (1034)	2.53% (1.70-3.75)	0.45 (0.29-0.71)		0.68 (0.42-1.10)	
	**Attended STI clinic, last 5 years**					<.001		.14
		Yes	1342 (779)	7.4% (5.9-9.4)	1		1	
		No	3865 (2855)	3.90% (3.27-4.63)	0.51 (0.37-0.69)		0.76 (0.53-1.09)	
	**STI service use, last year** ^f^					.02		.61
		Yes	1908 (1130)	5.80% (4.65-7.22)	1		1	
		Not reported	3366 (2552)	4.08% (3.39-4.90)	0.69 (0.51-0.94)		1.10 (0.77-1.58)	
	**STI** ^g^ **diagnosis, last 5 years**					.75		.14
		No	4830 (3419)	4.65% (4.03-5.36)	1		1	
		Yes	398 (225)	4.2% (2.4-7.3)	0.91 (0.50-1.64)		0.63 (0.35-1.16)	

^a^ Unweighted N=5312, weighted N=3703. Denominators vary due to item nonresponse.

^b^ Denominator restricted to those aged 17 and older. No academic qualifications: left school at age 16 without passing any exams/gaining any qualifications (excludes qualifications gained at an older age); academic qualifications typically gained at age 16: left school at 16 having passed some exams/gained some qualifications (eg, English General Certificate of Secondary Education [GCSE] or equivalent); studying for or attained further academic qualifications: left school at age 17 or older.

^c^ Based on National Statistics Socioeconomic Classification (NS-SEC) code. No job: no job of ≥10 hours per week in the last 10 years.

^d^ Quintile of adjusted Index of Multiple Deprivation for Great Britain.

^e^ Defined as reporting STI clinic attendance within the last year or responses other than “Internet” for questions listed in Table 1 within the last year. Exceptions (non-Internet responses which were ignored) were (1) where participants had indicated friend, parent/relative, or partner as sources of contraceptive supplies (free-text response) and (2) where participants had selected “family member/friend,” “self-help books/information leaflets,” “self-help groups,” and “have not sought any help” as sources of advice/help about their sex life.

^f^ Defined as reporting any of: STI clinic attendance, chlamydia testing, or HIV testing within this last year.

^g^ Natsal definition of STIs excludes thrush.

#### Sexual Behavioral Factors

Use of Internet information/support was more commonly reported by women (but not men) reporting multiple sexual partners in the last year and among both men and women reporting new sexual partners in the last year, but these associations disappeared after age adjustment. Among women (but not men), use of Internet information/support was more commonly reported by those who reported multiple sexual partners in the previous year with whom they had not used condoms (AOR 1.90, 95% CI 1.11-3.26, *P*=.03). Men reporting sex with another man in the previous 5 years were more likely to report use of Internet information/support (AOR 2.44, 95% CI 1.27-4.70, *P*=.008), whereas no association with same-sex sex in the previous 5 years was observed among women. Men and women reporting seeking sexual partners online within the previous year were more likely to report use of Internet information/support than those not reporting seeking partners in this way (men: AOR 1.80, 95% CI 1.16-2.79; women: AOR 3.00, 95% CI 1.76-5.13).

#### Sexual Health Care Use

No association was observed between reporting use of Internet information/support and reporting STI diagnosis or diagnoses in the past 5 years. Use of Internet information/support was more common among those reporting recent non-Internet sources of sexual health care and advice/help, and having attended an STI clinic in the last 5 years, but not after adjusting for age. No association was observed with having used STI services in the previous year.

#### Preference for Internet Sources of Diagnosis/Treatment of Sexually Transmitted Infections and Condoms/Contraception

Less than 2% of sexually experienced participants aged 16 to 44 years reported that the first place they would look for diagnosis/treatment if they suspected that they had an STI would be an Internet site offering treatment. Among sexually experienced persons aged 16 to 44 years reporting use of any contraceptive method in the previous year, 5.45% men and 1.14% women indicated they would prefer to obtain supplies from an NHS or Department of Health website (Table 5).

**Table 5:** Preference for Internet sources of diagnosis/treatment of sexually transmitted infections and condoms/contraception.

Header	Men		Women	
	N, unweighted (weighted)	% (95% CI)	N, unweighted (weighted)	% (95% CI)
Would first look on an Internet site offering treatment for diagnosis/treatment if STI suspected^a^	3589 (3668)	1.77% (1.27-2.46)	5269 (3670)	0.81% (0.57-1.14)
Preferred source of contraceptive supplies would be NHS/Dept of Health website^a^	2793 (2743)	5.45% (4.52-6.56)	4116 (2781)	1.14% (0.82-1.58)

^a^ Question wording, response options, and eligible respondents are detailed in Table 2.

## Discussion

### Principal Findings

Although Internet access is nearly universal in Britain, data from a recent national probability sample survey show that use of the Internet for key sexual health reasons is rare in the British population. Specifically, prevalence of reported use of Internet STI services is very low and reported use of the Internet for condoms/contraceptive supplies is also uncommon, particularly among women. Reporting recent use of Internet information and support websites for help/advice about one’s sex life was slightly higher, especially among younger people and among those who reported higher sexual risk behavior, including MSM and people who sought sexual partners online. However, those using information/support websites for advice/help with their sex lives may be from populations typically considered to have better access to sexual health care: the better-educated, residents of certain urban areas, and (among men) those of higher socioeconomic status. Despite this potential social inequality, those who reported recent use of information/support websites were as likely to report at least one previous STI diagnosis as those who did not report this.

### Findings in Relation to Other Studies

We know of no other studies that have estimated the prevalence of use of the Internet for sexual health reasons or identified associated factors in a nationally representative sample. The association we found between use of information/support websites for advice/help with one’s sex life, and younger age, is unsurprising given young people’s greater Internet use [26], smartphone ownership [27-29], and greater need for sexual health care indicated by levels of reported STI diagnoses and STI clinic use [16,17]. Research on the acceptability of using the Internet to deliver conventional sexual health services reveals similar findings with respect to age [30-32] and education [33].

Differences in men’s and women’s sexual behaviors [34,35] and health-seeking behaviors [36-38] are well-documented, but our study revealed little difference by sex in the prevalence of reported use of information/support websites for advice/help with one’s sex life (although there were some differences in associations observed among men and women, and more men than women reported that they would first look online for diagnosis/treatment if they suspected that they had an STI). In the English chlamydia screening program, the NCSP, more tests are performed among young women than among young men [5], perhaps due to women’s greater engagement with contraceptive and other health services where screening is offered. Women also account for a greater proportion of tests in the NCSP’s Internet-ordered home-sampling services, but with less discrepancy by gender compared to other NCSP testing venues (with the exception of military settings) [5]. In our study, use of the Internet for condoms/contraceptive supplies was reported by more men than women, perhaps reflecting gendered norms about who obtains condoms [39].

Surveys of patients attending genitourinary medicine (GUM) clinics in England, conducted almost a decade before Natsal-3, found patients reporting Internet sex seeking were also more likely to report use of the Internet for sexual health information [40], similar to the association we observed between Internet sex seeking and use of information/support websites.

Echoing our study’s finding, little difference was found by IMD quintile in the proportion of NCSP Internet-ordered chlamydia home-sampling kits returned (2010) [5]. However, we found no studies using NCSP data to compare demographic or behavioral characteristics of those using Internet-ordered kits with the wider population in the target age range. Although Internet-based sexual health services have been viewed as a promising way of reaching rural populations, we found relatively low use of information/support websites in these areas.

### Strengths and Limitations

Use of Natsal-3 data has allowed our analyses to examine a wide range of sociodemographic, behavioral, and health service use variables, in a sample representative of the resident British population, in relation to use of information/support websites for advice/help with one’s sex life. Despite survey data being self-reported and, therefore, subject to recall and social desirability biases, they are of high quality; use of CASI was demonstrated to facilitate reliable reporting of sensitive information [41] and cognitive testing of several survey modules maximized the likelihood of questions being interpreted as intended [42]. Furthermore, the survey’s response rate was similar to that achieved for other major social surveys undertaken in Britain at that time [43,44] and item nonresponse was typically very low [18,19]. Importantly, in this rapidly evolving field, we focused on reported behavior in the year before the survey interview and Natsal-3 data are relatively recent (collected 2010-2012). However, there may have been changes in norms regarding Internet use for sexual health since data collection.

The very low prevalence of most outcome variables examined meant that their associations could not be explored. The exception was reported use of the Internet for advice/help with one’s sex life, but even this was reported by less than 5% of the study population; therefore, rare behaviors could not be included as explanatory variables in the analysis. We adjusted only for age in the multivariable analysis. Due to small numbers in some subgroups, we had to treat some variables crudely (eg, ethnicity), creating categories large enough to obtain sufficient subgroup sizes. This limits explanatory potential; for example, we cannot explore differences between black Caribbean and black African ethnicities. The subgroup mixed/Chinese/other is not particularly meaningful, although creation of this category gave sufficient subgroup sizes to explore associations with Britain’s major ethnic groups (Asian, black, white).

Natsal-3 survey questions (Tables 1 and 2) serve various purposes and were not designed for our particular study. We cannot be sure about how questions were interpreted. Our main outcome variable (use of information/support websites for advice/help with one’s sex life) was based on responses to a question located in the survey module entitled “Sexual Function.” However, we assumed that the question was interpreted more broadly than about sexual function alone because “sexual function” was neither mentioned in the question nor visible on the computer screen at the time, and sex life was defined broadly (see Methods). Supporting our assumption, we found that among sexually active persons aged 16 to 44 years who reported use of information/support websites at this question, more than half agreed that they felt satisfied with their sex life, more than half disagreed that they felt distressed or worried about it, and more than two-thirds disagreed that they had avoided sex because of sexual difficulties (their own or a partner’s; data not shown). This suggests that many who reported use of Internet help/advice with their sex life were doing so for reasons other than sexual function problems, although we cannot be sure. In terms of applicability of our findings to sexual health broadly defined [1], our variable may not have captured use of the Internet in relation to all aspects of sexual health, such as support and counseling following nonvolitional sex. It seems unlikely that participants would have considered this type of service use to be help/advice with their sex life, although perhaps they would if nonvolitional sex occurred in the context of a sexual relationship.

An advantage of our study is that we were able to consider those who had used the Internet for a range of sexual health reasons and also those who would prefer to use it for sexual health care, although we lack data on which particular websites were used/preferred. However, the low proportions who reported a preference for using the Internet for STI diagnosis/treatment, or a preference for accessing contraception from an NHS website, probably underestimate the proportions that might choose Internet-based services if they were well-regulated and based in the NHS. This is because relevant survey questions (Table 2) each allowed a single response and provided no description of the Internet services, which might be difficult for respondents to envisage or assumed to be costly because such services are not currently available through the NHS. The question also specified “if an STI was suspected”: in this context, a consultation with a health care professional may seem most appropriate, whereas for a routine STI check-up, Internet services might hold greater appeal. Given how common it has become to look up symptoms and health information online before contacting a health professional, we believe that responses to the STI diagnosis/treatment question might poorly reflect the proportion that would use an Internet-ordered test if they found a reputable service offering this during their online search.

We acknowledge that even an ideal survey question cannot give us a definitive answer about who will use online sexual health interventions and services in the future. However, we feel our main outcome variable, which addresses use of information/support websites (as distinct from lay advice/help sought online) for sexual health broadly defined, reflects those who may take up online sexual health services and interventions, fitting with their existing sexual health-seeking behavior.

### Implications for Policy and Practice

Low levels of use of the Internet for contraception and STI services may reflect the limited availability and quality of currently available online services—particularly at the time the data were collected (2010-12) and in relation to STI testing and treatment [3,5,45]. Also, many methods of contraception cannot feasibly or legally be provided online. Qualitative and quantitative research could explore awareness, expectations, and barriers to use of currently available online sexual health services.

Greater proportions reported use of information/support websites for advice/help with their sex lives, particularly among young people. This suggests scope for expansion of provision in the future, in this cohort and subsequent cohorts who have also grown up with the Internet, and as the range and quality of Internet sexual health care increase (as is likely given existing trends). An example of improved quality is the legalization and regulation of HIV home testing in the United Kingdom, available online [10]. However, our study suggests that if use of Internet sexual health care followed patterns of online help/advice seeking, health inequalities might increase, especially if expansion of online sexual health care was coupled with reduced provision of conventional sexual health care. “Digital divides” by socioeconomic status have been widely documented [11], with eHealth a specific area of concern [46,47]. This study’s findings regarding education and socioeconomic status may reflect that Internet use is lower among those with less education and lower incomes [48]. Although home Internet access was high in the population of interest in Natsal-3, the survey did not ask about Internet use more generally, including via a personal device, which may vary across social strata. Having a smartphone or laptop/tablet might allow greater access to the Internet for sexual health than a household’s shared personal computer if privacy from other household members is important. Since the data were collected for Natsal-3 between 2010 and 2012, there have been further increases in smartphone ownership [49,50] and Internet access [51], which may reduce differences in proportions using the Internet for sexual health by socioeconomic status and/or education. However, if these differences relate to differences in health care-seeking behavior, inequalities may be more persistent. Research should examine these associations further and evaluations of new Internet-based interventions and services should monitor and model impacts on both on STI transmission and on health inequalities. Interventions may also be required to promote eHealth should groups be identified that have good Internet access, yet are underserved by online and conventional health care.

## Abbreviations

AOR: age-adjusted odds ratio
CAPI: computer-assisted personal interview
CASI: computer-assisted self-interview
GCSE: General Certificate of Secondary Education
GUM: genitourinary medicine
HIV: human immunodeficiency virus
IMD: Index of Multiple Deprivation
MSM: men who have sex with men
NHS: National Health Service
NS-SEC: National Statistics Socio-Economic Classification
OAC: Output Area Classification
STI: sexually transmitted infection
